# Detection of culprit presence in multiple-culprit crimes: A comparison of combined and separate lineup-presentation formats

**DOI:** 10.1371/journal.pone.0336456

**Published:** 2026-06-03

**Authors:** Ulla Lichtenhagen, Nicola Marie Menne, Raoul Bell, Carolin Mayer, Amelie Therre, Axel Buchner

**Affiliations:** Department of Experimental Psychology, Heinrich Heine University, Düsseldorf, Germany; Julius-Maximilians-Universitat Wurzburg, GERMANY

## Abstract

Although crimes involving multiple culprits are widespread, there is still a lack of understanding of how the police should construct lineups for multiple suspects in these cases. In most of the few guidelines that exist it is recommended to present multiple lineups separately in succession, with each lineup including one suspect. This replicates, for each suspect in a multiple-culprit case, the procedure used for a single suspect in a single-culprit case. However, this recommendation could be reconsidered if it turned out that, if multiple culprits are present in the lineups, the face of one culprit serves as a contextual facial cue that enhances the eyewitness’s ability to detect another culprit within the same display—an effect that could only occur if all lineups are presented together in a combined lineup-presentation format, allowing them to be visible concurrently. Two experiments using a staged-crime video with four culprits were conducted to test whether presenting simultaneous lineups combined as opposed to separately affects culprit-presence detection. In Experiment 1, it was tested whether culprit-presence detection differs between combined and separate lineup-presentation formats. No significant difference was found, indicating comparable performance between the two lineup-presentation formats. In Experiment 2, we compared a condition in which three culprit-present lineups were combined—allowing for multiple potential contextual facial cues—with conditions involving separate lineups and combined lineups with only one culprit-present lineup, where contextual facial cueing was theoretically not possible. Culprit-presence detection did not differ between the combined format with three culprit-present lineups and the other conditions. Given the absence of clear benefits of the combined-lineup-presentation format, a cautious, pragmatic conclusion is to continue presenting each suspect in a separate lineup, consistent with most current recommendations.

## Introduction

A notable number of crimes such as burglary, assault and robbery involve multiple culprits working together as a collective [see 1–8]. Statistics indicate that such multiple-culprit crimes account for up to one-third of all criminal cases [1,2,8]. Nevertheless, research on lineup construction for multiple suspects in multiple-culprit cases remains scarce, leaving law enforcement with little to no empirically grounded guidance on how to conduct lineups in these cases. While the procedure for conducting lineups involving a single suspect in a single-culprit case is well defined, most guidelines lack specific recommendations for handling lineups involving multiple suspects in a multiple-culprit case [9–13]. In the few guidelines that exist, the predominant recommendation is to present one lineup for each individual suspect and to present these lineups separately—one at a time—so that an eyewitness gives a response for one lineup before seeing the lineup for the next suspect [14–16]. However, there are exceptions. According to Tupper et al. [2], the manual for identification procedures in the Netherlands includes the recommendation to present photographic lineups for multiple suspects together in a combined format rather than each lineup separately. Whereas presenting lineups for multiple culprits separately has the advantage of simply extending the standard procedure for a single suspect in a single-culprit case to multiple suspects in a multiple-culprit case, a deviation from the separate-lineup format should be based on a strong rationale. One theoretical rationale for such a deviation could be the potential benefit of contextual facial cueing. Based on prior work on contextual effects in face recognition [cf. [17–19]] it can be hypothesized that the concurrent presence of multiple culprits when lineups are presented in a combined format could provide recognition-enhancing contextual retrieval cues, thus increasing the chances of detecting culprits in lineups. We will henceforth refer to this as the contextual-facial-cueing hypothesis.

On the one hand, multiple-culprit crimes require the processing of more faces which makes the identification of culprits more challenging compared to single-culprit crimes [20–23]. On the other hand, it is theoretically possible that the concurrent presence of multiple culprits could provide contextual retrieval cues that facilitate face recognition. Presenting the lineups created for each suspect together in a combined format (e.g., Lineup 1 consisting of Suspect 1 and *n* fillers matched to Suspect 1 combined in one single display with Lineup 2 consisting of Suspect 2 and *n* fillers matched to Suspect 2) rather than separately (e.g., Lineup 1 first and Lineup 2 only after the response to Lineup 1) would allow the eyewitness to view all suspects and fillers concurrently. If the suspects presented in a combined lineup are in fact the culprits, each culprit’s face may serve as a contextual facial cue facilitating the detection of another culprit within the same display. To evaluate the contextual-facial-cueing hypothesis, the present experiments were conducted to test whether culprit-presence detection is influenced by presenting multiple lineups combined rather than separately, using the well-validated two-high threshold (2-HT) eyewitness identification model [24,25].

At first glance, presenting lineups of multiple suspects together may appear to conflict with the basic principle that only one suspect should be included in each lineup [26]. This principle was introduced to ensure that a lineup does not consist solely of suspects but also includes a sufficient number of known-to-be-innocent fillers so that if eyewitnesses make a guessing-based selection, they are more likely to select a filler than a suspect [27]. This mechanism serves to protect suspects from being selected based on guessing, at least to some degree. Presenting multiple lineups in a combined format does not disrupt this protective mechanism, as long as each suspect remains accompanied by the same number of fillers, as in the example given in the previous paragraph. The protection against guessing-based selection is preserved because the probability of selecting a suspect by chance remains inversely proportional to lineup size. If the combined format enhances culprit-presence detection via contextual facial cueing, then the combined format could therefore be justified within existing procedural safeguards.

Interestingly, it has been reported that recognition of a target face improves when it is presented alongside another face with which it was previously encoded, compared to when it is presented alone or with a new face [e.g., [17,19]]. This effect can be explained by context reinstatement, where the paired face serves as a contextual facial cue that enhances the recognition of the target face [18]. Applied to eyewitness identification, this suggests that when multiple culprits are encoded together as part of the same crime event, each culprit’s face could serve as a contextual facial cue that supports the detection of another culprit’s face. Thus, presenting lineups for multiple suspects in a combined format might enhance culprit-presence detection by recreating part of the context in which the culprits were originally seen, compared to presenting the lineups separately one after another. However, findings on contextual facial cueing in face recognition are mixed: some results show benefits [17,19], while others do not [18]. At a theoretical level, it has been argued that in recognition tasks, the target stimulus itself is a highly effective retrieval cue that may already provide sufficient access to the encoding episode, leaving little room for additional contextual retrieval cues to exert an effect [28,29]. Whether or not the presence of another culprit in a combined lineup-presentation format provides a benefit for culprit-presence detection thus remains an open empirical question.

While in most previous studies, each suspect was presented in a separate lineup [e.g., 30], alternative lineup formats for multiple suspects in multiple-culprit cases have been explored in two prior studies. In both studies the effects of the lineup-presentation format were measured in terms of observable eyewitness identification performance [6,31]. In one study, Hobson and Wilcock [6] introduced a novel format for presenting the lineups of three suspects. Participants (*N* = 72) initially viewed three sequential lineups—one for each suspect—without giving a response. The same lineups were then shown again in a reverse order, at which point the participants were required to give their responses. This novel sequential lineup-presentation format was compared to the standard sequential lineup-presentation format in which participants gave their responses immediately upon the first presentation of the lineups. The culprit-identification rates were higher in the novel sequential lineup-presentation format compared to the standard sequential lineup-presentation format. However, these findings do not inform the evaluation of the contextual-facial-cueing hypothesis because the lineups were presented separately rather than combined, implying that the suspects were never viewed concurrently.

In the other study, Wells and Pozzulo [31] introduced the *two-person-serial-lineup* format as a novel approach for conducting lineups in crimes involving two culprits. This format merges two sequential lineups—one for each suspect—into a single unified presentation. Each photograph from the lineup for the first suspect was presented next to a photograph from the lineup for the second suspect. Participants (*N* = 150) viewed these pairs sequentially, giving a response for each pair before proceeding to the next pair until all photographs were shown. The authors hypothesized that the presence of one face might enhance the eyewitness’s ability to detect the other face. However, the two-person-serial-lineup format did not produce a higher culprit-identification rate than standard sequential or simultaneous lineup formats. Although each photograph was presented alongside a photograph from the other lineup, the two suspects were never shown together; instead, a suspect was always paired with a filler. Thus, it remains unclear whether the lack of an effect was due to the suspects not being presented together concurrently, potentially preventing contextual facial cueing from occurring.

**Fig 1:**
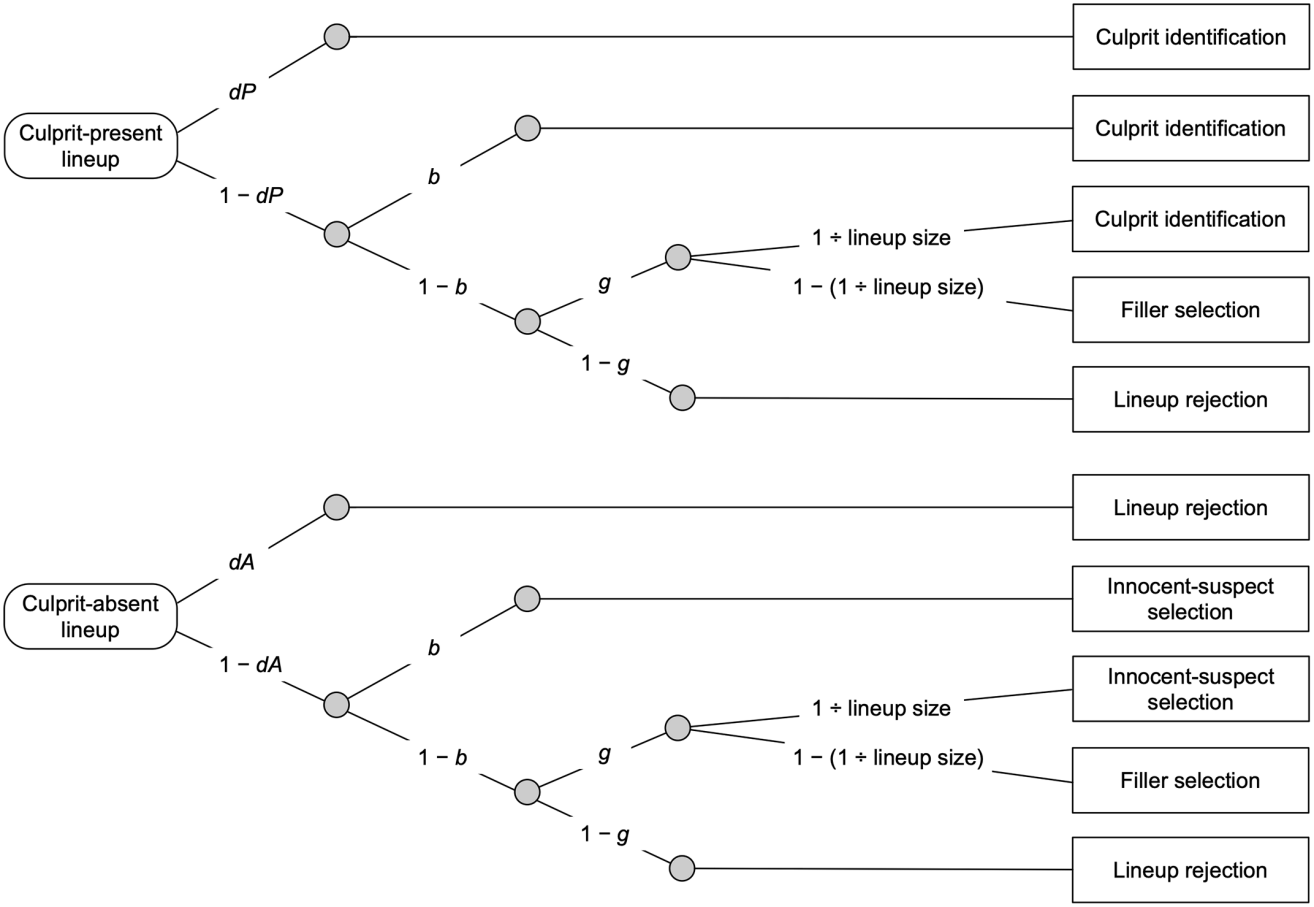
The 2-HT eyewitness identification model. The 2-HT eyewitness identification model is illustrated in the form of processing trees. The rounded rectangles on the left represent the two types of possible lineups: culprit-present lineups and culprit-absent lineups. Letters along the branches denote the parameters that represent the latent cognitive processes (*dP*: probability of culprit-presence detection; *dA*: probability of culprit-absence detection; *b*: probability of biased suspect selection; *g*: probability of guessing-based selection). The sampling probability of randomly selecting the culprit (upper tree) or the innocent suspect (lower tree) if guessing-based selection occurs is given by 1 ÷ lineup size. Rectangles on the right represent the resulting observable response categories.

Another important consideration is that in previous studies the focus was solely on observable behavior. In other words, the conclusions regarding eyewitness performance were derived from raw response rates. This approach has a key limitation: observable behavior can originate from different latent cognitive processes [32,33]. For instance, if an eyewitness correctly identifies a culprit from a lineup, it is unclear which latent cognitive process led to this particular response. The eyewitness may have indeed detected the culprit’s presence in the lineup, but it is also possible that the identification was merely the result of a guess. To separately measure the latent cognitive processes underlying observable eyewitness responses, a suitable measurement model is needed. Here we utilized the 2-HT eyewitness identification model [24,25] which belongs to the family of multinomial processing tree models [32,33]—a class of straightforward and highly accessible measurement models for which easy-to-use free software is available [34], as are comprehensive tutorials [35] which cover aspects such as model specification, parameter estimation and hypothesis testing. As previously explicated [24,25,36–46], this measurement model allows to estimate the probabilities of two detection-based processes (culprit-presence and culprit-absence detection) and two non-detection-based processes (guessing-based selection and biased suspect selection) that may occur when eyewitnesses are presented with a lineup (see explanations below). Parameter estimation is based on all six response categories available in lineups: correct culprit identifications, false selections of innocent suspects, false and correct lineup rejections and filler selections in culprit-present lineups and culprit-absent lineups. The 2-HT eyewitness identification model is illustrated in Fig 1.

The upper tree in Fig 1 illustrates the latent cognitive processes that may occur when a culprit is present in the lineup. The presence of the culprit is detected with probability *dP*, resulting in a culprit identification. This process is sensitive to manipulations that affect, for example, how clearly and how long the culprit’s face was visible at encoding [24,25]. Longer exposure durations and more favorable viewing conditions are associated with higher probabilities of culprit-presence detection, whereas poorer encoding conditions are associated with lower probabilities of culprit-presence detection. If the presence of the culprit is not detected, which occurs with probability 1 − *dP*, the culprit can still be selected as a consequence of non-detection-based processes. With probability *b*, biased suspect selection occurs. Parameter *b* captures the probability that a suspect is selected for reasons unrelated to memory because the suspect’s face or photograph stands out from the fillers. This process is determined by the fairness of the lineup [24,25,36,39]. If the lineup is unfair and the suspect noticeably stands out, biased suspect selection occurs with probability *b* > 0. This process is sensitive to manipulations that affect, for example, how distinctive the suspect’s photograph appears relative to the filler photographs at test [24,25]. For instance, lineups in which filler photographs are digitally morphed may be associated with a higher probability of biased suspect selection because morphed photographs often contain subtle artifacts that can cause the non-morphed suspect photograph to stand out compared to lineups in which no photographs are morphed [36]. It has been shown empirically that the effects of lineup unfairness are captured by this parameter, thereby permitting the remaining model parameters (*dP*, *dA*, and *g*) to be estimated independently of, and without being contaminated by, unfairness-related influences [24,25]. In case of no biased suspect selection, which occurs with the conditional probability 1 − *b*, participants may still select a lineup member based on guessing with probability *g*. Guessing-based selection is sensitive to manipulations that affect, for example, eyewitnesses’ expectations as to whether there is a high or a low probability that the culprit is present in the lineup [24,25] Instructions implying a high probability of culprit presence are associated with high probabilities of guessing-based selection, whereas instructions implying a low probability of culprit presence are associated with low probabilities of guessing-based selection. Guessing-based selection leads either to the selection of the culprit with the random sampling probability that is equal to 1 ÷ lineup size or to the selection of a filler with the complementary probability 1 − (1 ÷ lineup size). To illustrate, in a six-person lineup, a guessing-based selection will result in a selection of the culprit with a probability of 1 ÷ 6 and of a filler with probability 5 ÷ 6, illustrating that “a witness who chooses randomly is far more likely to land on a filler than the suspect” [27, p. 11]. With probability 1 − *g*, participants do not make a guessing-based selection and the lineup is falsely rejected.

The lower tree in Fig 1 illustrates the processes that may occur when a culprit is absent from the lineup, implying that the suspect is innocent. The absence of the culprit is detected with probability *dA*, resulting in a rejection of the lineup. This process is sensitive to manipulations that affect how easily a culprit-absent lineup can be rejected. Some culprit-absent lineups make it easier to determine that none of the lineup members is the culprit. For example, when all lineup members in a culprit-absent lineup—including the innocent suspect—share a conspicuous facial feature such as a large birthmark that the culprit did not have, it becomes obvious that none of the lineup members matches the culprit. This increases the probability of detecting the culprit’s absence and results in higher *dA* estimates compared to lineups in which the detection of the absence of the culprit is more difficult [25]. If the absence of the culprit is not detected, which occurs with probability 1 − *dA*, the same non-detection-based processes as in culprit-present lineups may occur. With probability *b*, biased suspect selection leads to the selection of the innocent suspect. No biased suspect selection occurs with probability 1 − *b*. In this case, participants may still make a guessing-based selection with probability *g*, resulting in either the selection of the innocent suspect with probability 1 ÷ lineup size or the selection of a filler with probability 1 − (1 ÷ lineup size). If participants do not make a guessing-based selection, which occurs with probability 1 − *g*, the lineup is correctly rejected.

The validity of the 2-HT eyewitness identification model has been demonstrated extensively both in dedicated validation experiments [25] and in reanalyses [24] of data from a wide range of studies conducted in different laboratories with different procedures and stimulus materials [47–54]. These successful validations underscore the robustness and generalizability of the model parameters. Importantly, all model parameters have been shown to sensitively reflect the cognitive processes they were designed to measure. We can thus be sure that, for instance, the assessment of culprit-presence detection is uncontaminated by lineup fairness, guessing-based selection, or the detection of the absence of the culprit.

The present experiments serve to test whether presenting simultaneous lineups for multiple suspects in a combined format or separate format affects the probability of culprit-presence detection. If multiple culprits are present in the lineups, the contextual-facial-cueing hypothesis implies that displaying the lineups in a combined format should enhance the eyewitness’s ability to detect the presence of the culprit. This is so because, according to the contextual-facial-cueing hypothesis, the face of one culprit should serve as a contextual facial cue for another culprit. In Experiment 1, we used the procedure from previous studies [25,36–45] where participants viewed two culprit-present lineups and two culprit-absent lineups. If contextual facial cueing occurs, then the presence of one culprit’s face should enhance the detection of the other culprit’s presence in the combined lineup-presentation format. The culprit-presence-detection parameter *dP* should thus be higher for combined lineups than for separate lineups. In Experiment 2, we compared a condition in which three culprit-present lineups were combined—allowing for multiple potential contextual facial cues—with conditions involving separate lineups and combined lineups with only one culprit-present lineup. If contextual facial cueing occurs, then the culprit-presence-detection-parameter *dP* should be higher in the condition with three combined culprit-present lineups compared to the control conditions where contextual facial cueing was theoretically not possible.

Beyond culprit-presence detection, it is also important to examine whether combining lineups influences guessing-based selection. Although there is no clear a priori hypothesis about how lineup-presentation format might affect guessing, such an effect cannot be ruled out. For instance, participants may feel greater pressure to select someone in each lineup when the lineups are presented separately, as they may feel uncomfortable letting multiple separate opportunities to identify someone from the lineup pass. Conversely, in the combined format, participants may experience a different type of pressure, as they may perceive this as their only opportunity to choose someone. Given these possibilities, it is crucial to use a measurement model that can distinguish between detection-based and guessing-based selection processes, a key strength of the 2-HT eyewitness identification model [24,25].

## Experiment 1

### Method

#### Participants.

Participants were recruited using the Horizoom research panel (www.horizoom-panel.de), a panel certified under ISO 20252 which ensures rigorous quality control. Of the 903 datasets collected from participants who completed the sociodemographic questionnaire at the beginning of the experiment, 114 were excluded because participants either did not complete the experiment or revoked their consent to use their data, 13 were excluded because participants failed the attention check (described in the *Materials and procedure* section) and 10 were excluded due to duplicate participation. The final sample, characterized by a diverse level of education, included the data of 766 participants (360 female, 405 male, 1 non-binary) aged between 18 and 79 years (*M* = 42, *SD* = 14; one reported age was excluded from the descriptive statistic due to an apparent typing error). In Experiment 1, one between-subjects independent variable was manipulated: the lineup-presentation format (combined vs. separate). Participants were randomly assigned to one of the two conditions: the combined lineup-presentation format (*n* = 382) and the separate lineup-presentation format (*n* = 384). Consistent with previous research [25,36–38,40–43,45] we used G*Power [55] to perform a sensitivity analysis which was based on a χ² goodness-of-fit test, given that the goodness-of-fit statistic *G*² used for the model-based statistical tests is asymptotically χ²-distributed [56]. The sensitivity analyses showed that, given error probabilities of α = β = .05 (and thus a statistical power of 1 – β = .95) and a sample size of *N* = 766 with four responses per participant, it was possible to detect an effect of the lineup-presentation format (combined vs. separate) on culprit-presence detection (*df* = 1) of size *w* = 0.07.

#### Ethics statement.

The ethics committee of the Faculty of Mathematics and Natural Sciences at Heinrich Heine University Düsseldorf has granted ethical approval for the experiments reported here. The experiments were conducted in compliance with the Declaration of Helsinki. All participants provided informed consent electronically by selecting an online checkbox prior to the start of the experiment. All participants were adults aged 18 years or older. No minors took part in the study. At the beginning of both experiments, participants were warned that they would see a video including physical and verbal violence. Participants were informed that they had the option to withdraw from the study if they preferred not to watch such a video. At the end of the experiments, participants were debriefed that the crime had been staged for research purposes.

#### Materials and procedure.

The materials and procedure were essentially as described in previous experiments [25,36–38,40–43,45,46] with the exception of the manipulation of the lineup-presentation format described below. The experiment was conducted online and was implemented in *SoSci Survey* [57] (www.soscisurvey.de). Participants were only allowed to use a desktop or laptop computer to take part; the use of tablets or smartphones was not possible. On the welcome page of the study, participants were asked to switch their browser window to full-screen mode. After having given their informed consent, participants reported their age, gender and their highest level of formal education.

*Staged-crime videos.* Next, participants were randomly assigned to watch one of two staged-crime videos (henceforth referred to as Video 1 and Video 2). In both videos, four hooligans of the German soccer club FC Bayern München (the culprits) attacked a fan of the rival club Borussia Dortmund (the victim) at a bus station. Participants had a clear view of the culprits’ faces from all angles including the frontal view. The victim and the culprits wore shirts and scarves showing their club’s colors and logo. The culprits insulted the victim, mocked him and tossed his personal belongings around. As the video progressed, the culprits became physically more aggressive. They pushed the victim around until he got knocked to the ground. The four culprits carried on with their verbal and physical abuse until they suddenly noticed another person approaching (not shown in the video). They then swiftly ran away. Both videos showed the same events in the same sequence and timing, but featured different actors. However, the actors were selected such that the victim of Video 1 resembled the victim of Video 2 and each of the four culprits in Video 1 had a high resemblance to a corresponding culprit in Video 2 in terms of body shape, hair color and hairstyle (i.e., Culprit A in Video 1 resembled Culprit A in Video 2, Culprit B in Video 1 resembled Culprit B in Video 2 and so on). The videos lasted approximately 130 seconds and were presented at a resolution of 885 × 500 pixels. Participants started the video by pressing a ‘Start’ button. Participants could not fast-forward, replay or pause the video and were only able to proceed to the next page after having watched the whole video.

*Attention check.* To ensure that the video had been attended, a 10-alternatives attention-check question followed, asking participants to select the option that correctly identified the type of person shown in the video (correct response: soccer fans). Data of participants who had failed the attention check were excluded from data analyses, as described in the Participants section above.

*Lineup* *instructions.* After the attention-check question, participants were instructed that they would see several lineups featuring a series of faces and that their task was to identify the FC Bayern München hooligans previously seen in the video if present. Participants were explicitly informed that each row represented one lineup. They were also advised that any particular lineup may or may not contain a previously seen face, emphasizing the importance of both correctly identifying the culprit and rejecting the lineup if the culprit is absent.

*Photographs.* Each lineup included the facial photographs of one suspect and five fillers. For each lineup, five white male filler faces aged between 18 and 29 years were taken from the Center for Vital Longevity Face Database [58]. Fillers were chosen based on their resemblance to each respective pair of culprits from the two parallel videos (e.g., to Culprit A shown in Video 1 and to Culprit A shown in Video 2) in terms of body shape, hair color and hairstyle by two of the present authors. The photographs of both suspects and fillers showed a frontal view of the face and neck against a black background. All faces had neutral facial expressions. All photographs were edited to harmonize face sizes and lighting conditions and were created in a resolution of 142 × 214 pixels. The photographs were automatically scaled and adjusted to match the screen resolution, ensuring consistent presentation across conditions and allowing all presented faces to be visible on the participant’s computer screen (as mentioned above, participation with tablets or smartphones was not possible) at the same time.

**Fig 2:**
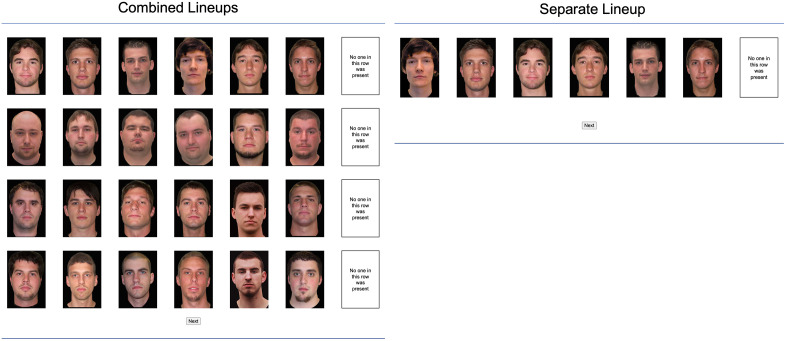
Example of combined and separate lineup-presentation formats. On the left, four lineups are displayed concurrently in the combined lineup-presentation format. On the right, a single lineup is shown in the separate lineup-presentation format. In both conditions, each lineup contained five fillers and one suspect. To the right of each lineup, an option labeled ‘No one in this row was present’ allowed participants to reject the respective lineup. Participants proceeded with the study by pressing the ‘Next’ button. The figure shows English translations of the German response options shown to the German participants. We have written consent of the person representing the suspect to show the photograph generated for the experiments. The photographs of the fillers were taken from the Center for Vital Longevity Face Database [58], which is freely available for academic researchers.

*Lineup construction and presentation.* Four lineups were presented. Two of the four lineups were randomly selected to be culprit-present lineups, while the other two were culprit-absent lineups. As in previous studies [25,36–46 the crossed-lineup procedure was used. In culprit-present lineups, the suspect was the culprit from the video the participant had previously seen. In culprit-absent lineups, a designated innocent suspect was included in the lineup, rather than arbitrarily assigning this role to one of the fillers. The designated innocent suspect was one of the actors from a video the participant had not seen. For instance, if a participant had seen Video 1, then two culprits from Video 1 (e.g., Culprit B and Culprit D) served as the culprits in the culprit-present lineups, while two culprits from Video 2 (in this example, Culprit A and Culprit C) served as innocent suspects in the culprit-absent lineups. This crossed-lineup procedure is similar, but not identical, to the single-lineup procedure proposed by Oriet and Fitzgerald [59]. In the single-lineup procedure, a single lineup is shown to all participants and the suspect’s guilt is determined by which of two videos participants saw (either featuring the suspect from the lineup or a similar-looking person). As suggested by Quigley-McBride and Wells [60] it is advisable to additionally determine randomly for each participant whether a suspect appears in a culprit-present or culprit-absent lineup. This randomization ensures that each suspect has an equal likelihood of being presented as a culprit or as an innocent suspect (see [61], for an early implementation of this technique), which in turn allows for systematic variation in culprit presence versus absence across multiple lineups and ensures that no systematic differences exist between photographs of culprits and innocent suspects. This is so because any such differences are balanced out: Individuals who serve as culprits for some participants serve as innocent suspects for others, such that the same photographs appear in both roles across participants. The fillers are therefore equally well matched to both the culprits and the innocent suspects. Moreover, the crossed-lineup procedure is particularly suitable for cases involving multiple culprits, as it allows for variation across lineups in whether a culprit or an innocent suspect is presented, as in the present study. The order of the lineups and the positions of all photographs in a lineup, including the positions of the photographs of the culprit and the innocent suspect, were determined randomly. Examples of combined and separate lineups can be found in Fig 2.

Irrespective of the lineup-presentation format, there was one lineup for each suspect. Each lineup consisted of the suspect’s photograph and five photographs of fillers. In line with common procedures in U.S. law enforcement [62] and eyewitness research [for instance [21,30,31], we constructed each lineup using one suspect and five fillers, resulting in a total of six lineup members. The photographs within each lineup were shown simultaneously, arranged in a horizontal row. Participants could identify a person as a culprit by clicking on one of the photographs in the row of photographs of a lineup. The selected photograph was then highlighted by a colored frame. Participants could reject the lineup by selecting the ‘No one in this row was present’ option displayed to the right of the row of photographs of the lineup. This response option was displayed in a rectangular frame corresponding in size to the frames of the photographs. The frame of this response option was also highlighted with a colored frame if the option was selected. Participants could revise their response to a lineup by clicking on another photograph in the lineup or on the ‘No one in this row was present’ option pertaining to the lineup.

*Combined lineups.* In the combined lineup-presentation format, the four simultaneous lineups were arranged in rows on a single page: the first lineup at the top, followed by the second, the third and finally the fourth at the bottom. The photographs were automatically scaled to fit the participant’s computer screen (as mentioned above, participation with tablets or smartphones was not possible) such that all four lineups were visible at the same time in four rows of six images each, allowing participants to view all 24 photographs concurrently. Participants had to give a response for each lineup by selecting one of the photographs in each row or by rejecting the lineup using the ‘No one in this row was present’ option. Pressing the ‘Next’ button was possible only after a response had been provided to each of the four lineups and took participants to the final page of the experiment.

*Separate lineups.* In the separate lineup-presentation format, the four lineups were constructed in the same manner as in the combined lineup-presentation format. However, each of the four lineups was presented on a separate page, requiring participants to give a response for the first lineup before it was possible to see the second lineup on the subsequent page and so on. Pressing the ‘Next’ button was possible only after a response had been provided to the current lineup and brought participants to the next lineup unless they had already responded to the fourth lineup in which case they were taken to the final page of the experiment.

*Debriefing*. On the final page of the experiment, participants were debriefed and informed of the fact that the crime shown in the video had been staged for research purposes. Participants were also given the opportunity to revoke the consent to the use of their data and they were thanked for their participation. Following a click on the ‘Finish study’ button, participants were redirected to the panel provider where they were compensated for their participation.

### 3.1. Results

For all analyses reported in this article, parameter estimates and goodness-of-fit tests were calculated using *multiTree* [34]. The α level was set to .05. The observed response frequencies for Experiment 1 are reported in Table 1. To analyze the data of Experiment 1, two instances of the model depicted in Fig 1 were needed, one for the combined lineup-presentation format and one for the separate lineup-presentation format.

**Table 1:** Response frequencies and proportions for culprit-present and culprit-absent lineups as a function of lineup-presentation format.

	Combined lineups	Separate lineups
**Culprit-present lineups**		
Culprit identifications	284 (.37)	271 (.35)
Filler selection	180 (.24)	200 (.26)
Lineup rejections	300 (.39)	297 (.39)
**Culprit-absent lineups**		
Innocent-suspect selections	108 (.14)	86 (.11)
Filler selection	257 (.34)	242 (.32)
Lineup rejections	399 (.52)	440 (.57)

**Note.** Observed response frequencies and proportions (in parentheses) for culprit identifications, filler selections and lineup rejections in culprit-present lineups and innocent-suspect selections, filler selections and lineup rejections in culprit-absent lineups as a function of lineup-presentation format (combined lineups vs. separate lineups).

To obtain a testable base model and maintain consistency with previous applications of the 2-HT eyewitness identification model [24,25,36,–42, 45], we constrained the biased-suspect-selection parameter *b* and the culprit-absence detection parameter *dA* to be equal across the conditions. The term 1 ÷ lineup size, representing the probability of randomly sampling the suspect if guessing-based selection occurs, was set to 0.16667 (as an approximation to 1 ÷ 6). The base model incorporating these constraints fit the data, *G*²(2) = 5.12, *p* = .077, which supports the assumptions incorporated into the base model, including the constraints that neither biased suspect selection nor culprit-absence detection differ across conditions. This was to be expected, given that the same sets of suspect and filler photographs were used in both conditions, resulting in comparable lineup fairness, and that there was no a priori reason to assume differences in the probability of detecting the absence of the culprit across lineup-presentation formats. Parameter *b* was estimated to be 0.06 (*SE* = 0.01). This value is slightly above zero, indicating that the lineups were not perfectly fair. However, this does not pose a problem for any of the model-based analyses because the biased-suspect-selection parameter *b* has been shown empirically to capture lineup unfairness, ensuring that the other model parameters are measured without being contaminated by lineup unfairness [24,25,36,39]. Parameter *dA* was estimated to be 0.03 (*SE* = 0.04).

**Fig 3:**
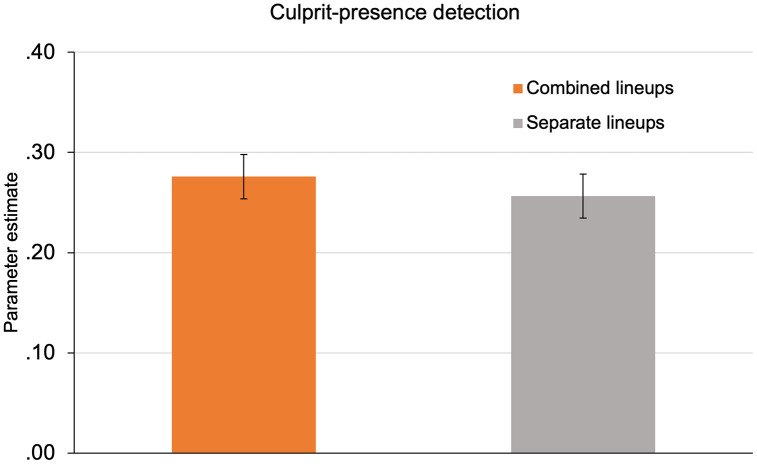
Estimates of culprit-presence detection by lineup-presentation format. Estimates of parameter *dP* representing the probability of culprit-presence detection as a function of lineup-presentation format (combined lineups vs. separate lineups). The error bars represent standard errors.

The estimates of the culprit-presence-detection parameter *dP* are displayed in Fig 3. One advantage of multinomial processing tree models is that hypothesis tests can easily be performed directly at the level of the parameters representing the postulated cognitive processes. For instance, the hypothesis that the probability of culprit-presence detection differs between the combined and the separate lineup-presentation format can be tested by setting parameter *dP* to be equal between the lineup-presentation formats. This constraint generates one degree of freedom. If the fit of the model including this additional constraint is significantly worse than the fit of the base model not including this constraint, then it can be concluded that the probability of culprit-presence detection differs between the lineup-presentation formats in the direction indicated by the parameter estimates. However, there was no significant difference in the probability of culprit-presence detection as a function of whether the lineups were presented in the combined or in the separate lineup-presentation format, ∆*G*^2^(1) = 0.47, *p* = .491. The hypothesis that the combined lineup-presentation format improves culprit-presence detection relative to the separate lineup-presentation format thus has to be rejected. As a side note, there was also no significant difference in the probability of guessing-based selection between the combined and the separate lineup-presentation format, ∆*G*^2^(1) = 0.60, *p* = .438, indicating that lineup-presentation format did not influence guessing-based selection. Parameter *g* was estimated to be 0.44 (*SE* = 0.02) for the combined lineup-presentation format and 0.42 (*SE* = 0.02) for the separate lineup-presentation format.

### 3.2. Discussion

In Experiment 1, we used the procedure from previous studies [25,36–38,40–43,45,46] where participants viewed two culprit-present lineups and two culprit-absent lineups. This design allowed us to test whether the concurrent presence of one culprit’s face in the combined lineup-presentation format enhances the detection of the other culprit’s presence compared to the separate lineup-presentation format. The model-based analysis showed that culprit-presence detection was comparable across combined and separate lineup-presentation formats, leading to the rejection of the contextual-facial-cueing hypothesis, even though the large sample size ensured a high sensitivity to detect a contextual-cueing effect if it existed.

In Experiment 2, we compared a condition in which three culprit-present lineups were combined—allowing for multiple potential contextual facial cues—with conditions involving separate lineups and combined lineups with only one culprit-present lineup, where contextual facial cueing was theoretically not possible. This three-versus-one lineup structure was chosen because it maximizes the contrast between conditions while maintaining model identifiability. Participants were assigned to one of the following conditions: (1) Combined lineup-presentation format with three culprit-present lineups and one culprit-absent lineup, (2) separate lineup-presentation format with three culprit-present lineups and one culprit-absent lineup, (3) combined lineup-presentation format with one culprit-present lineup and three culprit-absent lineups, and (4) separate lineup-presentation format with one culprit-present lineup and three culprit-absent lineups.

When three culprit-present lineups are presented in the combined lineup-presentation format, the faces of two culprits could potentially serve as contextual facial cues for detecting the presence of the other culprit. In contrast, no contextual facial cueing is expected when the separate lineup-presentation format prevents the three culprits from being seen concurrently. Furthermore, if only one culprit-present lineup is presented, then no contextual facial cueing is possible regardless of whether the faces are presented in the combined or separate lineup-presentation format, as there are no other culprits who could serve as contextual facial cues. Therefore, the to-be-tested hypothesis is whether parameter *dP* is higher for the combined lineup-presentation format with three culprit-present lineups compared to all other conditions grouped together (i.e., the separate lineup-presentation format with three culprit-present lineups or one culprit-present lineup, and the combined lineup-presentation format with one culprit-present lineup).

## Experiment 2

### 4.1. Method

#### Participants.

Participants were recruited using the Horizoom research panel (www.horizoom-panel.de). Of the 893 datasets collected from participants who completed the sociodemographic questionnaire, 87 were excluded because participants either did not complete the experiment or revoked their consent to use their data, 9 were excluded because participants failed the attention check and 16 were excluded due to duplicate participation. The final sample, characterized by a diverse level of education, included the data of 781 participants (371 female, 409 male, 1 non-binary) aged between 18 and 93 years (*M* = 42, *SD* = 15). Two between-subjects independent variables were used in Experiment 2: (1) lineup-presentation format (combined vs. separate) and (2) number of culprit-present lineups (three vs. one). Participants were randomly assigned to one of four conditions: combined lineups with three culprits present (*n* = 197), separate lineups with three culprits present (*n* = 200), combined lineups with one culprit present (*n* = 192) and separate lineups with one culprit present (*n* = 192). A sensitivity analysis with G*Power [55] showed that, given error probabilities of α = β = .05 (and thus a statistical power of 1 – β = .95) and a sample size of *N* = 781 with four responses per participant, it was possible to detect an effect of lineup-presentation format (combined lineup-presentation format with three culprit-present lineups vs. all other conditions) on culprit-presence detection (*df* = 1) of size *w* = 0.06.

### 4.2. Materials and procedure

Materials and procedure were identical to those of Experiment 1 except that the number of culprit-present lineups and culprit-absent lineups was manipulated: In conditions with three culprits present, three of the four lineups were randomly selected to be culprit-present lineups while the remaining lineup was a culprit-absent lineup. In conditions with one culprit present, only one of the four lineups was randomly selected to be a culprit-present lineup while the other three were culprit-absent lineups.

### 4.3. Results

The observed response frequencies for Experiment 2 are reported in Table 2. To analyze the data of Experiment 2, four instances of the model depicted in Fig 1 were needed, one for the combined lineups with three culprits present, one for the separate lineups with three culprits present, one for the combined lineups with one culprit present and one for the separate lineups with one culprit present.

**Table 2:** Response frequencies and proportions for culprit-present and culprit-absent lineups as a function of lineup-presentation format and number of culprit-present lineups.

	Three culprits		One culprit	
	Combined lineups	Separate lineups	Combined lineups	Separate lineups
**Culprit-present lineups**				
Culprit identifications	237 (.40)	229 (.38)	66 (.34)	76 (.40)
Filler selection	137 (.23)	147 (.25)	50 (.26)	54 (.28)
Lineup rejections	217 (.37)	224 (.37)	76 (.40)	62 (.32)
**Culprit-absent lineups**				
Innocent-suspect selections	23 (.12)	29 (.15)	59 (.10)	68 (.12)
Filler selection	65 (.33)	54 (.27)	167 (.29)	195 (.34)
Lineup rejections	109 (.55)	117 (.59)	350 (.61)	313 (.54)

**Note.** Observed response frequencies and proportions (in parentheses) for culprit identifications, filler selections and lineup rejections in culprit-present lineups and innocent-suspect selections, filler selections and lineup rejections in culprit-absent lineups as a function of lineup-presentation format and number of culprit-present lineups (combined lineups, three culprits vs. separate lineups, three culprits vs. combined lineups, one culprit vs. separate lineups, one culprit).

The same constraints as in Experiment 1 were applied to obtain a testable base model, that is, parameters *b* and *dA* were each set to be equal across the four conditions. The base model incorporating these constraints fit the data, *G*^2^(6) = 4.01, *p* = .675. The estimates of parameters *b* and *dA* were 0.06 (*SE* = 0.01) and 0.13 (*SE* = 0.05), respectively.

**Fig 4:**
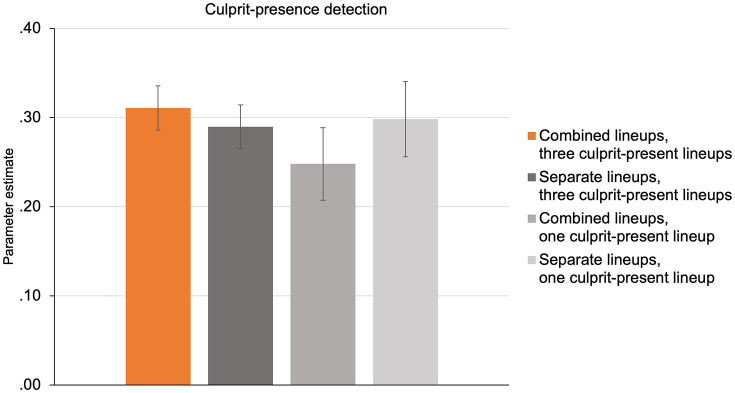
Estimates of culprit-presence detection by lineup-presentation format and number of culprit-present lineups. Estimates of parameter *dP* representing the probability of culprit-presence detection in the combined lineup-presentation format with three culprit-present lineups, the separate lineup-presentation format with three culprit-present lineups, the combined lineup-presentation format with one culprit-present lineup and the separate lineup-presentation format with one culprit-present lineup. The error bars represent standard errors.

The estimates of culprit-presence-detection parameter *dP* are displayed in Fig 4. From a theoretical perspective, contextual facial cueing should be absent in conditions where at most one culprit is visible at any one time such that no other culprits can serve as contextual facial cues. This applies to the separate lineup-presentation formats, where each culprit-present lineup is presented individually and it applies to the combined lineup-presentation format with only one culprit-present lineup, where no other culprits are available to serve as contextual retrieval cues. It thus follows on a priori theoretical grounds that culprit-presence detection should not differ among the separate lineup-presentation format with three culprit-present lineups, the combined lineup-presentation format with one culprit-present lineup and the separate lineup-presentation format with one culprit-present lineup. To formally test this, parameter *dP* was restricted to be equal across these three conditions. As expected, the equality constraint was compatible with the data, ∆*G*^2^(2) = 0.99, *p* = .609, and was thus incorporated into a new base model, which fit the data, *G*^2^(8) = 5.01, *p* = .757.

This result provided the conditions necessary to test the key hypothesis derived from the contextual-facial-cueing hypothesis, namely that the combined presentation of three culprit-present lineups—where contextual facial cueing could theoretically occur if it existed—would lead to higher culprit-presence detection compared to all other conditions where such effects should not occur. As it turned out, culprit-presence detection in the combined lineup-presentation format with three culprit-present lineups did not significantly differ from culprit-presence detection in the three other conditions grouped together, ∆*G*^2^(1) = 0.90, *p* = .344. The contextual-facial-cueing hypothesis thus needs to be rejected.

A secondary analysis showed that guessing-based selection did not differ among the four conditions, ∆*G*^2^(3) = 6.70, *p* = .082. Parameter *g* was estimated to be 0.45 (*SE* = 0.02) for the combined lineup-presentation format with three culprit-present lineups, 0.44 (*SE* = 0.02) for the separate lineup-presentation format with three culprit-present lineups, 0.42 (*SE* = 0.03) for the combined lineup-presentation format with one culprit-present lineup and 0.50 (*SE* = 0.03) for the separate lineup-presentation format with one culprit-present lineup.

### 4.4. Discussion

In Experiment 2, a condition in which three culprit-present lineups were combined—allowing for multiple potential contextual facial cues—was compared with conditions involving separate lineups or combined lineups with only one culprit-present lineup such that contextual facial cueing was theoretically impossible. Yet even under these conditions, no contextual-facial-cueing effect was observed. Culprit-presence detection did not differ significantly between the combined lineup-presentation format with three culprit-present lineups and all other conditions. What is more, this result was observed despite the large sample size ensuring high sensitivity to detect potential differences among conditions if they existed. The results of Experiment 2 thus reinforce the conclusions from Experiment 1: Presenting lineups in a combined format does not improve culprit-presence detection. Consequently, these findings allow us to reject the contextual-facial-cueing hypothesis.

## General discussion

Crimes involving multiple culprits are widespread, yet research on best practices for conducting lineups involving multiple suspects in multiple-culprit cases is limited. Practical guidelines either lack specific recommendations for handling lineups involving multiple suspects in multiple-culprit cases [9–13] or the predominant recommendation is to present separate lineups, one for each suspect [14–16], following the standard approach used for performing a lineup with a single suspect in a single-culprit case to ensure the independent evaluation of each suspect (but see [2]). Here we evaluated an alternative—the combined lineup-presentation format—based on the hypothesis that viewing multiple culprits concurrently might enhance culprit-presence detection through contextual facial cueing. Before revising established procedures, it must be empirically demonstrated that combined lineups improve culprit-presence detection, while also ensuring that such a format does not increase guessing-based selections. The 2-HT eyewitness identification model [24,25] is particularly well-suited for addressing this question, as it separates detection-based processes from guessing-based selection, providing a comprehensive assessment of the cognitive mechanisms underlying eyewitness responses.

In Experiment 1 (*N* = 766), we used the procedure from previous studies [25,36–38,40–43,45,46] in which participants viewed two culprit-present lineups and two culprit-absent lineups. This allowed us to test whether the concurrent presence of one culprit in the combined lineup-presentation format enhances the detection of the other culprit’s presence compared to the separate lineup-presentation format. This hypothesis was disconfirmed by the results of Experiment 1. Culprit-presence detection did not differ significantly between the combined and separate lineup-presentation formats. This was so despite the large sample size which ensured a high sensitivity to detect differences if they existed.

In Experiment 2 (*N* = 781), we compared a condition in which three culprit-present lineups were combined—making multiple contextual facial cues available—with conditions involving separate lineups or combined lineups with only one culprit-present lineup, such that contextual facial cueing could not occur. Even under these conditions, culprit-presence detection remained comparable across all lineup-presentation formats. These findings reinforce the conclusions of Experiment 1: Culprit-presence detection does not benefit from a combined lineup-presentation format where multiple culprit-present lineups are presented concurrently.

One possible explanation for the absence of contextual-facial-cueing effects is that in recognition tasks, the target stimulus itself serves as a highly effective retrieval cue to its previous presentation, leaving little room for additional contextual retrieval cues to influence memory [28,29]. This may account for the mixed results found in studies on contextual facial cueing in face recognition [17–19].

Additionally, integration of information across lineups may be impaired**,** as face-space theory [63] suggests that faces are encoded individually based on physical features rather than contextual associations. This may lead to independent processing of each face, thereby reducing the likelihood that faces from different lineups are conceptually linked. One might also argue that presenting the lineups in rows, as in the combined format, encouraged participants to treat each lineup as a separate decision context, further limiting integration across lineups. However, this arrangement was necessary to give participants the option to reject individual lineups, which is a central feature of fair identification procedures.

Another important finding is that guessing-based selection did not differ between lineup-presentation formats. In other words, presenting separate lineups does not encourage guessing-based selections any more, but also not less, than presenting lineups in a combined format. This is a crucial finding, as guessing leads to the identification of a culprit and an innocent suspect with the same probability, implying that only detection-based identifications but not guessing-based identifications can provide meaningful evidence for evaluating a suspect’s potential guilt. It is thus undesirable if a suspect is identified based on guessing. The present findings thus offer some reassurance that presenting lineups separately does not increase the risk for suspects to be selected based on guessing.

A potential limitation of the present study is that it remains unclear whether contextual facial cueing might emerge in crimes involving an even larger number of culprits. However, Tupper et al. [2] surveyed police officers from Sweden, Belgium and the Netherlands about multiple-culprit crimes, and 94% indicated that such crimes typically involved only two to three perpetrators (p. 997). Thus, while future research could explore whether contextual-facial-cueing effects become more pronounced with a greater number of culprits, the practical relevance of such conditions may be limited. As a further limitation of this study, we did not assess confidence, which prevents us from drawing conclusions about potential effects of combining lineups for multiple suspects on confidence judgments.

## Conclusion

Since there was neither an increase in culprit-presence detection nor a decrease in guessing-based selection in the combined lineup-presentation format relative to the separate-lineup-presentation format, there is no empirical basis for preferring combined lineups over separate lineups involving multiple suspects in multiple-culprit cases. However, more research is needed before firm practical recommendations can be made. That said, a procedural advantage of the separate lineup-presentation format is that it allows well-established guidelines for presenting a lineup involving a single suspect in a single-culprit case to be directly applied to presenting lineups involving multiple suspects in a multiple-culprit case. In light of this, a cautious, pragmatic conclusion is to continue presenting each suspect separately, in line with most existing recommendations [e.g. 14–16].

## Supporting information

S1 File Labels for experimental conditions, data and equations. (PDF)
