# Supplementary material for: Detection of culprit presence in multiple-culprit crimes: A comparison of combined and separate lineup-presentation formats
Source: PLoS One. 2026 Jun 3;21(6):e0336456. doi: 10.1371/journal.pone.0336456 (PMC13232940; doi:10.1371/journal.pone.0336456)
Supplement: S1 File — (PDF) [file pone.0336456.s001.pdf]

S1 File. Labels for experimental conditions, data and equations.

Experimental conditions

In all data files “Experiment_1_Response_Frequencies_Comb_Sep.txt” and “Experiment_2_Response_Frequencies_Comb_Sep_3_1_culprits.txt” and as well as the equation files “Experiment_1_Equations.eqn” and “Experiment_2_Equations.eqn” the experimental conditions are defined as follow:

All lines including “combined_*” correspond to the group that was presented with combined lineups with two culprits present.

All lines including “separate_*” correspond to the group that was presented with separate lineups with two culprits present.

All lines including “combined_3_culprits_*” correspond to the group that was presented combined lineups with three culprits present.

All lines including “separate_3_culprits_*” correspond to the group that was presented with separate lineups with three culprits present.

All lines including “combined_1_culprit_*” correspond to the group that was presented with combined lineups with one culprit present.

All lines including “separate_1_culprit_*” correspond to the group that was presented with separate lineups with one culprit present.

Data and equations for all analyses

The data file labeled “Experiment_1_Response_Frequencies_Comb_Sep.txt” contains the frequencies of the different response categories that are possible in lineups, aggregated across participants and lineups for each experimental condition in Experiment 1.

The data file labeled “Experiment_2_Response_Frequencies_Comb_Sep_3_1_culprits.txt” contains the frequencies of the different response categories that are possible in lineups, aggregated across participants and lineups for each experimental condition in Experiment 2.

The equation files labeled “Experiment_1_Equations.eqn” and “Experiment_2_Equations.eqn” contain the model equations of the 2-HT eyewitness identification model that specify how the model-implied probabilities are assumed to lead to the response categories in Experiment 1 and Experiment 2.

These files are needed for the model-based analyses with multiTree [1].

In the following, we explain the abbreviations used in these files:

*_tp: Culprit present

*_tp_hit: Correct culprit identification in culprit-present lineups

*_tp_miss: False Filler selection in culprit-present lineups

*_tp_rej: False lineup rejection of culprit-present lineups

*_ta: Culprit absent

*_ta_fa: False innocent-suspect selection in culprit-absent lineups

*_ta_miss: False filler selection in culprit-absent lineups

*_ta_rej: Correct lineup rejection of culprit-absent lineups

Lineup size: number of lineup members, six lineup members in all experiments

Examples:

dP_combined_3_culprits: *dP*-parameter (probability of culprit-presence detection) for the group that responded to combined lineups with three culprits present.

3_culprits_ta_miss: Number of false filler selections in culprit-absent lineups of the group that responded to lineups with three culprits present.

1. Moshagen M. multiTree: A computer program for the analysis of multinomial processing tree models. Behav Res Methods. 2010;42(1):42-54. doi: 10.3758/BRM.42.1.42.
